# Barriers and facilitators to digital health tool adoption for hypertension management: systematic review of qualitative studies

**DOI:** 10.1136/bmjopen-2025-116004

**Published:** 2026-06-12

**Authors:** Emily Motta-Yanac, Victoria Riley, Naomi J Ellis, Aman Mankoo, Christopher J Gidlow

**Affiliations:** 1 Centre for Health and Development (CHAD), University of Staffordshire, Stoke-on-Trent, England, UK; 2 School of Medicine, Keele University, Newcastle-under-Lyme, University Road, Staffordshire, UK; 3 Research and Innovation Department, St Georges Hospital, Midlands Partnership University NHS Foundation Trust, Stoke-on-Trent, UK

**Keywords:** Primary Health Care, Telemedicine, Disease Management, Digital Technology, Hypertension

## Abstract

**Abstract:**

**Objectives:**

Digital health interventions (DHIs) show considerable promise in supporting hypertension self-management by promoting preventative care and self-monitoring. While their efficacy is increasingly evident, the long-term uptake, acceptance and sustained engagement with these tools are frequently challenged by issues such as usability, trust and varying user experiences. This review aims to synthesise qualitative evidence to identify barriers and facilitators and the key factors that impact the adoption, acceptance and engagement with DHIs for hypertension self-management.

**Design:**

Systematic review of qualitative literature using thematic analysis following Cochrane’s qualitative and implementation methods guidance.

**Data sources:**

PubMed, PsycInfo, Web of Science and the Cochrane Library were searched in February 2025.

**Eligibility criteria for selecting studies:**

The searches included relevant qualitative and mixed-methods studies on the use of digital devices for hypertension management, which described the barriers and facilitators associated with these tools. We included studies published from 2015 to 2025 to capture relevant evidence. Only studies published in English with a qualitative approach were included.

**Results:**

From an initial 10 943 identified publications, 15 met our inclusion criteria, primarily originating from Europe and the USA, exploring diverse racial and ethnic group experiences. Our thematic synthesis revealed 7 analytical and 22 descriptive themes detailing barriers and facilitators encountered by patients with hypertension, healthcare providers (HCPs) and caregivers. These themes covered technology utilisation, design components, linguistic and cultural relevance, healthcare factors, trust and credibility and interpersonal interactions.

**Conclusion:**

Our analysis underscores that factors such as the usability, design and relevance of social support profoundly influence the uptake and acceptance of DHIs in hypertension self-management among patients, caregivers and HCPs.

**PROSPERO registration number:**

CRD42023480389.

**STRENGTHS AND LIMITATIONS OF THIS STUDY**

- The systematic review adhered to rigorous methodological standards, including a comprehensive search strategy and a quality appraisal process to minimise bias.
- It offers valuable insights into the complex interplay of factors influencing digital health intervention adoption and effectiveness in hypertension management.
- The heterogeneity of study designs and populations included in the review may limit the generalisability of the findings.
- The review included studies regardless of their specific findings and quality, although it notes this was necessitated by the small number of qualitative studies available.
- The small number of qualitative studies on the topic required a broad approach to capture emerging themes comprehensively, which could be seen as a limitation regarding the depth or specificity of included studies.

## Introduction

 The growing number of individuals with chronic illnesses increasingly strains healthcare systems.^1^ Healthcare services are emphasising preventative care and self-monitoring of long-term conditions. Self-monitoring and co-interventions have proven effective in reducing blood pressure (BP).^2^ Digital health interventions (DHIs), including telehealth and smartphone apps, hold the promise of making a difference from traditional BP management.^3 4^ However, user-friendliness, accessibility and data privacy remain critical for long-term success.^3^ Digital tools can present challenges for both patients and healthcare providers (HCPs).^5^ Assessing user experience and satisfaction is, therefore, a critical step in implementing digital strategies.^6^ While quantitative evidence has established that DHIs can lead to a significant reduction in BP,^3 4^ there remains a clinical-engagement gap in the real-world application of these tools.^4^ Current literature frequently prioritises clinical outcomes (ie, frequency of BP readings or medication adherence rates), while offering limited insight into the psychological and sociotechnical mechanics that sustain or inhibit user engagement over time.^2 4^ This gap in the literature is twofold. First, although DHIs are advertised for their potential to foster self-management, fewer commercially available global hypertension apps involve medical experts in their development, leading to a deficit in perceived credibility and safety among both patients and HCPs.^4^ Second, existing qualitative data is often fragmented, focusing on single-app evaluations or specific demographic cohorts.^4 5^ There is a need to synthesise these diverse qualitative findings to identify common motivational drivers and inhibitory factors that dictate whether a tool from a novel download becomes an integrated part of a patient’s long-term care routine.

Therefore, our review aimed to explore the barriers and facilitators to using DHIs in managing hypertension among end-users (caregivers, patients, HCPs). A comprehensive grasp of the variables influencing the acceptance and consistent use of these technologies is necessary to incorporate digital health interventions into hypertension self-management successfully.^7^

Therefore, our systematic review sought to consolidate qualitative evidence on the barriers and facilitators influencing the use of digital health interventions for self-management of hypertension.

## Methods

This review followed the guidance of the Cochrane Handbook for Systematic Reviews of Interventions,^8^ and followed the Preferred Reporting Items for Systematic Reviews and Meta-analysis (PRISMA).^9^ The review protocol was preregistered on the International Prospective Register of Systematic Reviews (PROSPERO; CRD42023480389).

### Search strategy

A systematic computerised search in PubMed, the Cochrane Library (CENTRAL; cochranelibrary.com/central), the Web of Science (WoS) and PsycInfo (Psychology and Behavioural Sciences) was conducted in October 2024 to identify studies published between January 2015 and January 2025.

The search strategy was developed using text keywords and the Medical Subject Headings (MeSH) from relevant reviews and recently published literature (online supplemental Text S1-S4).

### Selection criteria and data extraction

Studies were selected based on the following inclusion criteria: participants with a primary diagnosis of hypertension; age ≥18 years; focused on DHIs for self-management; and used a qualitative or mixed-methods design. We specifically excluded studies focusing on hypertension in pregnancy (eg, preeclampsia).

The screening process involved two stages: title/abstract review, followed by full-text screening by the lead reviewer. Additionally, a randomly selected 10% of records were independently screened by a third investigator (AM), with any discrepancies resolved through team discussions.

In this review, the term ‘caregivers’ refers specifically to informal caregivers (eg, family members, spouses or friends) who provide unpaid support to patients with hypertension. A standard data extraction form was used. We synthesised the numeric data on participants’ socioeconomic status (SES) indicators, sex and ethnicity proportions. Participants’ quotes and main findings were analysed.

### Patient and public involvement

Patients or the public were not involved in the design, or conduct, or reporting, or dissemination plans of our research.

### Quality appraisal

The assessment of the methodological rigour, credibility and relevance of the included studies was conducted using the Critical Appraisal Skills Programme (CASP) checklists^10^ for qualitative studies and the MMAT (Mixed Methods Approach Tool) for mixed-method studies.^10^

### Data analysis

We conducted a thematic synthesis using Thomas and Harden’s methods for the thematic synthesis of qualitative research.^11^ This consisted of three stages: (1) line-by-line analysis of the findings from the studies using NVivo software V.1.6.1, (2) identifying and organisation of primary themes, and (3) analysis of similar concepts in the primary themes underlying the use of DHIs for the management of BP.

## Results

### Study characteristics

**Figure 1:**
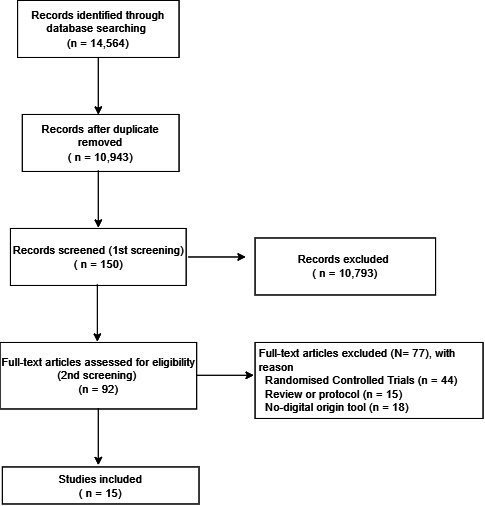
Preferred Reporting Items for Systematic Reviews and Meta-analysis (PRISMA) flowchart for the systematic review process.

Searches yielded 14 564 results, which were reduced to 10 943 after removing duplicates (figure 1). After the first screening, 92 were retrieved for full-text screening. As illustrated in the PRISMA flow diagram, 77 records were excluded during the full-text screening phase: 44 were randomised controlled trials (RCTs), 15 were review or protocol records and 18 did not use a digital-based tool. A total of 15 studies^12^,^26^ were included in this review. One study explored the feasibility of a phone-based platform,^23^ and two studies used SMS-phone-based^20 25^ for the intervention (online supplemental table S1). The remaining studies explored the use of phone-based apps. Two studies implemented an HCP (nurses, pharmacists) model to support the intervention.^25 26^

Most of the studies were conducted in the USA (33.3%), followed by the UK (13.5%). The remaining studies were undertaken in Ireland, Cambodia, China, Germany, Australia, Saudi Arabia and Nepal.

Among the included studies, high-quality methodology and relevant findings were reported in all the qualitative studies and mixed-method studies, except for one study by Culhane-Pera *et al*^14^ (online supplemental table S2 and online supplemental table S3). Culhane-Pera’s study assessed the acceptance of a mobile health model for hypertension self-management using a mixed-method approach. While the qualitative design adhered to the standard methods, the quantitative components compromised the overall quality.

### Baseline demographics

The participant sample comprised 55.5% females and 44.5% males, with an overall mean age of 56 years across the included studies (online supplemental table S1). Asian participants accounted for over half (57.1%) of the sample size, followed by white (32.9%) and then Latino (9.8%) participants. Other ethnic groups each represented 0.1% of the sample. Among the participants who reported SES indicators (66.7%), half of these studies reported that their participant samples were predominantly of high SES. This high-SES group was characterised by higher rates of employment, post-secondary education attainment and higher income levels. Meanwhile, 15.4% were of low SES, as indicated by unemployment or reliance on social welfare.

### Synthesis of qualitative findings: Barriers and facilitators using digital health interventions

**Figure 2:**
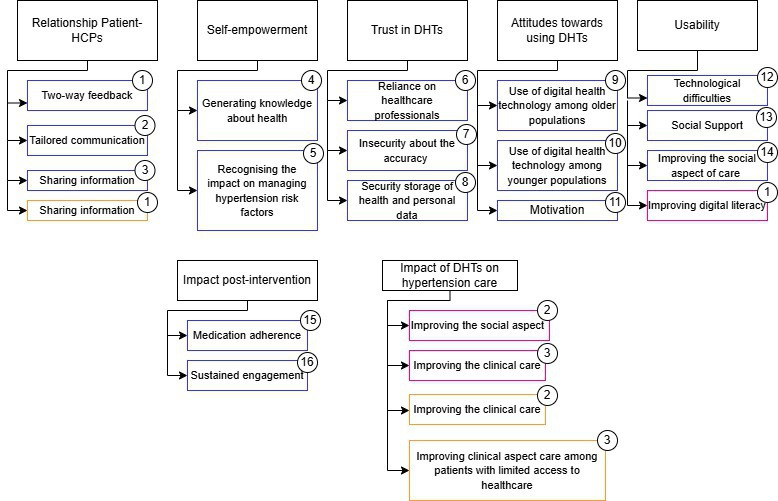
Thematic map illustrates analytical and descriptive themes related to DHTs in hypertension care from multiple end-users’ perspectives. Each analytics theme encompasses multiple descriptive themes depicted with connecting arrows. Each descriptive theme is colour-coded according to its end-user perspective. Blue themes represent the patient’s perspective, yellow boxes represent HCPs’ perspective and pink boxes represent caregivers’ perspective. Numbered circles indicate descriptive themes that correspond to their representation in figure 3. DHTs, digital health technologies; HCPs, healthcare providers.

**Figure 3:**
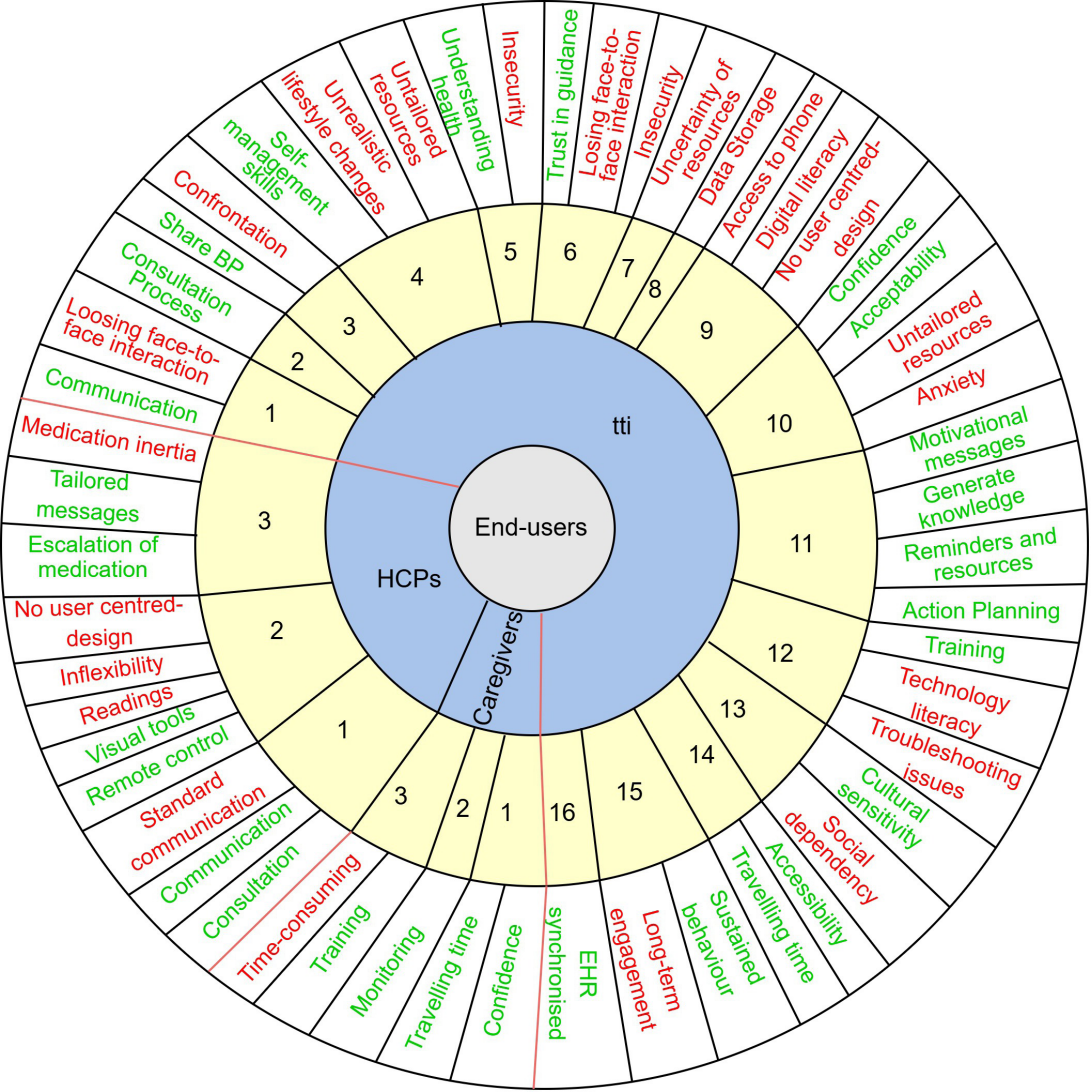
Circular diagram illustrates barriers and facilitators to the use of DHTs in hypertension care, mapped by end-user group. The inner circle identifies the end-user groups, with patients, HCPs and caregivers each occupying distinct sectors. The in-between ring is divided into 16 numbered segments, each representing a descriptive theme corresponding to those in figure 2. Within each segment, specific barriers and facilitators are listed in red and green, respectively, are shown in the outer ring. BP, blood pressure; DHTs, digital health technologies; EHR, electronic health record; HCPs, healthcare providers.

Initial thematic analysis grouped findings by end-user (patients, professionals, informal caregivers/family members) experiences with digital health interventions for hypertension. After an in-depth review, themes were synthesised and streamlined (online supplemental table S4), resulting in the final set of themes presented in online supplemental table S5. Key themes were developed through an iterative process, integrating facilitators and barriers. The analysis of the included studies identified seven analytical themes encompassing 21 descriptive themes (figure 2). Under each theme, both barriers and facilitators were identified (figure 3). Participant quotes are provided in the text to substantiate the data for each theme. More exemplary quotations are provided in online supplemental table S5.

#### Relationship between patient and HCPs

The theme explored how digital health tools influenced the patient–provider relationship and clinical consultations. Patients reported that two-way feedback; “…felt (like) I was put on the radar screen” (patient),^19^ and personalised messages improved consultations.^12^,^1419 21 22 24^ However, some patients feared losing face-to-face consultations^16 21^; “I’ve had nothing back, and nobody has asked to see me face to face, I suppose that really is a slight frustration, that you’re not getting much feedback from them” (patient).^16^ Despite this, some patients feared opening confrontations with practitioners.^12 17^

HCPs worried about standardised communication^15^; “You can’t just say, ‘Do this,’ which is fine. I mean, you have to understand that it’s just not going to be a quick (fix). I mean, it’s that relationship, right?”.^15^ However, they also acknowledged the potential for DHIs to enhance communication and provide richer patient data, although recognising that not all patients would benefit equally from such digital-first approaches.^15^

Patients and HCPs perceived digital tools as bridging the communication gap and fostering better collaboration. For patients, DHIs serve as a supplementary tool rather than a replacement for traditional consultations, suggesting that effective dialogue is crucial for improving the clinical encounter for both patients and providers. HCPs expressed concern over standardised communication that might overlook individual patient nuances and preferences.

#### Self-empowerment of patients and caregivers through the use of digital health tools

Empowering patients and caregivers through digital health tools can lead to improved health outcomes and a greater sense of control over their healthcare journey.^27^

Digital health tools offer significant potential for empowering patients and caregivers by enabling them to generate knowledge about their health,^12^,^1618 19 23 24 26^ and fostering a critical understanding of high BP risk factors^12^,^1619 24^; “…and I look how it goes and how my blood pressure level developed and draw my conclusions from that” (patient).^24^

However, unrealistic expectations of lifestyle changes and a lack of tailored messages were frustrating for patients^18^; “It didn’t really relate to me as I don't smoke” (patient)^18^*,* and limited access to relevant educational resources built barriers to engagement^18^; “Some patients may be literate but technological literacy is also another thing, like using an app whilst checking your BP” (HCP).^18^ How to use an app is a new challenge on its own, insecurity related to maintaining engagement and consistent use of digital tools also hindered self-empowerment^12^,^1619 24^; “The easiest (remote monitoring digital health tool) to use but there are several steps involved” (patient).^15^

This theme suggests that DHTs provide opportunities for self-empowerment by enabling patients and caregivers to gain knowledge and a critical understanding of health management. This leads to an improved sense of control and can contribute to better health outcomes and clinical decision-making. However, unrealistic expectations, non-tailored messaging and difficulties maintaining engagement can hinder self-empowerment. Therefore, personalised approaches and user-friendly designs are essential to maximise the benefits of DHIs.

#### Trust in digital health technologies

The analytical theme of trust encompasses trust in digital health technologies, security and privacy and credibility. A foundational element of trust in DHTs stems from patients’ reliance on healthcare providers as credible sources; their guidance facilitates trust in the technology itself.^12^,^1419 20 24^ As one patient stated; “If messages are sent by you and others like you (health care professional), I will happily accept it”.^20^ However, this trust was undermined by a lack of face-to-face consultation,^12 19^ a lack of self-trust in engaging with behaviour-change content,^12 16^ insecurity about the accuracy of clinical outcomes^12 16 19 25^ and doubts about the secure storage of health and personal data.^12 22 24^ Concerns about data privacy are particularly salient in the digital age. As one patient expressed, “I am afraid if the apps share my details with other third parties, including Facebook”.^22^

Fostering trust in digital health technologies requires a multifaceted approach that considers the interplay between patient–provider relationships, data security, and patient self-efficacy.

#### Attitudes towards using digital health technologies

Motivation, accessibility and perceived benefits influenced attitudes towards using DHTs, which varied based on age and individual circumstances. Among younger populations, a sense of enhanced confidence in managing BP and increased acceptability of remote monitoring often led to more positive attitudes^14 24^; “I downloaded it and, yes, it was also easy to use” (patient).^24^ Addressing the barriers and needs of diverse age groups is essential for promoting positive attitudes towards using digital health technologies.

In contrast, for older adults, the adoption and acceptance of DHTs present a more complex picture. Barriers such as a lack of access to a phone, reduced digital literacy and a perceived lack of user-centred design can contribute to negative attitudes.^13 14 21 23 26^ Standardised messages and educational information also played a significant role, particularly when technology increases anxiety^12 19 22 25 26^; “Seeing my blood pressure readings at home makes me stressed sometimes” (patient).^25^ This highlights a critical consideration: DHTs should be designed not only to provide information but also to alleviate anxiety and promote a sense of well-being.

#### Usability of digital health technologies for patients and caregivers

Usability of DHTs is a multifaceted concept influenced by technical aspects, user skills and the social dimensions of technology. Patients and caregivers expressed that enabling training and readily available digital technical support can empower users to overcome technological difficulties associated with digital tools^1518^,^20 26^; “Future training should include recommendations on how to troubleshoot technological challenges”.^18^ However, a lack of user-friendly design was a significant barrier to access; “The SMS language should be simple and easy to understand for us” (patient).^20^ HCPs expressed a similar concern on the implementation of health management in digital tools; “How can we manage those mobile services? It will be challenging to implement”.^20^ The effectiveness of training is inextricably linked to digital literacy and the ability to troubleshoot issues independently.^12 14 15 18 19^

Social support emerged as a critical enabler in navigating usability challenges, with family and community support playing a vital role in facilitating technology use.^1314 18^,^21 24 25^ A key facilitator in improving the social aspect of usability is the increased access to healthcare in rural areas, enabled by technology^13 18 19 22 26^; “I think that my kids can help me use it, but they are very busy” (patient).^14^ A key facilitator in improving the social aspect of usability was the easy access to BP readings; “When I am asleep they (friends) would knock on my door to check their BPs” (patient).^18^ This support was mostly expressed among patients in rural areas.

#### Post-intervention adherence impact of the intervention

Sustained behaviour changes instilled during the intervention are crucial for post-intervention adherence. A key facilitator is maintaining health-related practices (eg, medication adherence).^18 19 26^ Although maintaining long-term engagement with digital tools poses a significant barrier,^18 22^ patients mentioned that synchronising electronic health records with BP readings could facilitate long-term engagement.^18 22^ One comment; “It’s like you are being watched by some health team and your BP is being managed”,^18^ illustrates the dual nature of this approach. On the one hand, it can foster a sense of accountability and encourage continued engagement. On the other hand, it can also raise concerns about privacy and autonomy.

#### Impact of digital technologies on hypertension care

DHTs offer a mixed bag of benefits and challenges for both healthcare providers and caregivers involved in hypertension care. For HCPs, these can improve clinical care by enabling remote BP monitoring and facilitating medication escalation.^13 15 17 20 23 26^ While this review focuses on qualitative experiences, these perceptions are supported by quantitative evidence demonstrating the clinical efficacy of DHIs. Recent meta-analyses of RCTs indicate that digital interventions significantly reduce office systolic BP by 3.21–4.05 mm Hg.^28^

However, some HCPs harbour reservations about standardised communication protocols and a perceived lack of flexibility in implementing BP reading plans^13 17^; “You’ve got a plan and now that’s changing and now do I have to make another three-point plan? And that’s really irritating and now I’ve gone off piste”.^17^

For caregivers, these technologies may increase confidence in providing care^18^ and reduce travel time to health facilities^13 18^; “Even if our BP is high we wouldn’t know unless we come to the clinic but that wasn’t the case when we had the machines”.^18^

However, as mentioned in the reported concerns, DHTs may inadvertently increase caregiving responsibilities, and the complexities in device operation could present significant challenges for caregivers, potentially diminishing the overall utility of the technology.

## Discussion

We conducted a systematic review and synthesis of qualitative studies to explore the barriers and facilitators when using DHIs for self-management of hypertension. The limitation of our review lies in including studies regardless of findings and quality; however, the small number of qualitative studies on this topic necessitates a broad approach to capture emerging themes comprehensively. Despite this, the review followed rigorous methodological standards in qualitative synthesis, offering valuable insights into the complex interplay of factors influencing DHI adoption and effectiveness in hypertension management. Our review identified key themes, including the relationship between patients and HCPs, self-empowerment, trust, attitudes, usability, adherence and the impact on HCPs and caregivers. Our findings aligned with previous studies on digital health interventions, which have identified infrastructure and technical issues as significant barriers for HCPs.^29 30^ These findings also emphasised the need for user-friendly interfaces, tailored education and ongoing support to ensure effective engagement and adherence.^31^

The interplay between patients and HCPs significantly influenced the effective adoption of DHIs for hypertension management. Trust and guidance offered by HCPs emerged as key factors influencing patients’ trust and engagement with those technologies.^31^ This suggests the importance of fostering a strong patient–provider relationship to facilitate the adoption and effective use of digital tools for hypertension management. Our findings suggested that while some patients considered communication, feedback mechanisms and ongoing support from HCPs to be facilitators, some HCPs were concerned about standardised communication and a lack of flexibility in BP reading plans. This emphasises the need to build mutual trust and balance the benefits of human-digital interaction, ensuring that patient needs are addressed, while HCPs maintain sufficient care control. Simultaneously, these tools emerged as facilitators of patient and caregiver empowerment by enhancing their critical awareness of health management, fostering self-efficacy and promoting a patient-centred approach.^32^ Therefore, tailoring interventions to individual needs and preferences, providing ongoing support and education, and addressing concerns about data security and privacy to foster confidence and trust in the DHI technology.^33^

Usability was another critical factor, often presenting challenges related to technical difficulties^34^ and varying levels of digital literacy among users. However, social support emerged as a critical enabler in overcoming these hurdles. Emotional and informational assistance from family and community members played a key role in improving the use of DHIs. This underscores the importance of addressing both technical and social determinants of health to ensure equitable access and sustained engagement for all users.

Furthermore, the economic feasibility of digital health adoption emerged as a pivotal factor for real-world implementation. While clinical trials often mitigate financial barriers by providing free equipment and connectivity, our analysis suggested that for the general hypertensive population, the costs of smart devices and recurring mobile data charges remain potential barriers to adoption.

Beyond the immediate technical and financial barriers identified, these findings reveal a deeper tension between clinical utility and user-centred design. While devices often prioritise high clinical accuracy for medical approval, our synthesis suggested that this utility frequently comes at the cost of usability, where the burden of frequent monitoring leads to monitoring fatigue and eventual disengagement. This challenge is compounded by the unique, asymptomatic nature of hypertension. Unlike more symptomatic chronic illnesses, the ‘silent’ nature of high BP can lead users to perceive digital management as a low-priority task compared with more pressing daily demands, often resulting in forgetfulness or decreased motivation.^13 16^ Addressing this requires a shift in how these tools are conceptualised. An important insight from this qualitative evidence is the essential and often overlooked role of the ‘informal caregiver’. Family members and spouses often act as the primary facilitators for technology adoption, yet they remain largely marginalised in current patient-centric design and regulatory frameworks.^35^ As regulatory bodies increasingly emphasise ‘human factors’ and usability templates for device approval, there is a clear need to integrate these qualitative sociocultural insights to ensure that future technologies are not only clinically validated but are also sustainably integrated into the complex domestic and social environments of patients.

Maintaining long-term engagement with digital tools remained a significant barrier to the sustained adherence of DHIs.^36^ Achieving lasting improvements in hypertension control requires sustained behaviour changes and adherence to these interventions.^37^ Our review suggested that translating traditional patient support into interactive virtual components is complex. Further research is needed to determine the optimal balance between human and computerised support to foster sustained long-term behaviour change.^38^ However, studies suggest that designing user-friendly interfaces and addressing individual concerns and preferences facilitates sustained engagement.^39^

Beyond direct patient use, DHIs offer broader implications for HCPs and caregivers by supporting care coordination, health monitoring and shared decision-making at a system level.^40^ Our findings suggested these technologies can support HCPs in medication management and improve patient communication, though a few still voice concerns about overly standardised protocols and a perceived lack of flexibility in care plans. For caregivers, DHIs held the potential to boost their confidence in providing care and reduce the need for travel to healthcare facilities. However, the small existing literature on integrating caregivers into these interventions suggests a need for more focused attention on their needs to fully leverage DHIs and enhance the overall care process.^41^

Some limitations are present in this review. First, although a comprehensive search strategy was implemented across multiple databases following PRISMA guidelines, the review was restricted to studies focusing on digital-based tools, which may have excluded valuable data from hybrid health interventions that use older digital modalities. Second, the quality of the evidence was appraised using the CASP Checklist. While the included studies demonstrated high methodological rigour, a meta-synthesis is a secondary interpretation. The themes identified represent a synthesis of the authors’ interpretation of their participants’ experiences rather than an analysis of the raw data itself. Third, there is likely demographic and recruitment bias within the included studies. Studies that reported socioeconomic characteristics often found a high educational level among participants, which limits the generalisability of these findings to digitally marginalised or vulnerable populations facing more fundamental barriers to access.

## Conclusion

This synthesis aimed to identify the barriers and facilitators for access and engagement associated with using digital tools for hypertension self-management. Our findings revealed a lack of user-friendly design, data privacy concerns, a positive impact on tailored feedback, an eagerness to self-manage their health status and the need for social support and a human-like interface. Furthermore, the characteristics of individuals using digital tools for hypertension management included people living with hypertension and a willingness to engage in self-management of their condition. Personalised and tailored approaches that incorporate behaviour change techniques and address the needs of both patients and caregivers are crucial for enhancing the acceptability and long-term usage of these tools. HCPs considered DHTs great supplementary tools, while these impacted the quality of care and facilitated surveillance, the current design does not meet the needs of HCPs in terms of time-consuming data processing. Co-design methods involving all stakeholders can help identify the appropriate design features and intervention strategies to improve the impact of DHTs in hypertension management. Overall, this review highlights the need for user-centred design, accessible and affordable DHIs and targeted support for individuals with varying levels of digital literacy.

## Supplementary material

10.1136/bmjopen-2025-116004 online supplemental file 1

## Data Availability

All data relevant to the study are included in the article or uploaded as supplementary information.
