# Supplementary material for: Barriers and facilitators to digital health tool adoption for hypertension management: systematic review of qualitative studies
Source: BMJ Open. 2026 Jun 12;16(6):e116004. doi: 10.1136/bmjopen-2025-116004 (PMC13289156; doi:10.1136/bmjopen-2025-116004)
Supplement: online supplemental file 1 [file bmjopen-16-6-s001.docx]

Emily Motta-Yanac^1*^, Victoria Riley^1^, Naomi J. Ellis^1^, Aman Mankoo^1^, Christopher J. Gidlow^3,4^

^1^Centre for Health and Development (CHAD), Staffordshire University, Stoke-on-Trent, ST4 2DF, United Kingdon

^3^Keele University, School of Medicine, University Road, Staffordshire, ST5 5BG

^4^Midlands Partnership University NHS Foundation Trust, Research and innovation Department, St Georges Hospital, Corporation Street, Stafford ST16 3AG

^*^Corresponding Author at: College Rd, Stoke-on-Trent, ST4 2DE, United Kingdom

E-mail address: emily.mottayanac@research.staffs.ac.uk

Contents

1. Table S1: Summary of the characteristics of the studies included in the systematic review.
2. Table S2: The Critical Appraisal Skills Program (CAPS) checklist for qualitative studies.
3. Table S3: The Mixed Method Appraisal Tool (MMAT) for mixed-method studies.
4. Table S4: Streamlined codebook analytical-descriptive themes.
5. Table S5: Facilitators and Barriers, with Exemplar Quotations from All Participants.
6. Text S1: Search strategy in PubMed
7. Text S2: Search strategy in PsycINFO
8. Text S3: Search strategy in Cochrane Central
9. Text S4: Search strategy in Web of Science

Table S1. Summary of Findings (SoF) of DHI studies for management of high blood pressure. IG: Intervention Group, CG: Control Group, SES: socioeconomic status

| Study ID | Location | Ethnicity and | | | | | | | Principle |
| --- | --- | --- | --- | --- | --- | --- | --- | --- | --- |
|  |  | SES of participants; sample size | | | | | | | objective |
|  |  | Sample | SES (%) | Sex | | Age | | Ethnicity (%) |  |
|  |  | Size |  | Female (%) | Male (%) | Mean years age (years) | Group ages (years) |  |  |
| Glynn (2015) | Ireland | 50 | Geographic context:  Urban (48 %)  Rural (52%) | 50% | 50% | 59 | - | - | To explore peoples' views of digital tools for the self-management of hypertension and lifestyle behaviours |
| Nichols (2019) | Sub-Sahara  Africa | 31 | Geographic context:  Urban  IG (77.8 %)  CG (50%)  Rural  IG (22.8%)  CG (50%) | 33% | 67% | 54.3 | - | - | To explore peoples' and caregivers' perspectives on the post-mHealth intervention for hypertension management |
| Baratta (2022) | US | 31 | - | 56% | 44% | - | 30-39 (19%)   40-49 (38%)   50-59 (31%)   60-69 (13%) | White (50%)  Asian (31%) | To examine multi-stakeholders perceptions of hypertension management Humanwide to evaluate the program’s acceptability, appropriateness, feasibility, and sustainability. |
|  |  |  |  |  |  |  |  |  |  |
|  |  |  |  |  |  |  |  |  |  |
|  |  |  |  |  |  |  |  |  |  |
|  |  |  |  |  |  |  |  |  |  |
| Steinman (2020) | Cambodia | 70 | - | 60% | 40% | 55.4 | - | Asian (100%) | To understand the facilitators and barriers of chronic disease management and the acceptability, appropriateness, and feasibility of mHealth to support chronic disease management and strengthen community-clinical linkages to existing services. |
| Morton (2018) | UK | 35 | Education level:  Higher education (15%)  No formal education (15%)  A levels (15%) | IG: 71%  CG: 43% | IG: 29%  CG: 57% | 68 | - | White (98%)  Black (1%)  Pakistani (1%) | To explore the perceived burden and  The benefits of using a digital health intervention for self-managing BP were assessed using qualitative process interviews with intervention and usual care participants participating in a randomised controlled trial (RCT). |
|  |  |  |  |  |  |  |  |  |  |
| Morton (2021) | UK | 152 | - | 63% | 37% | - | - | - | To develop a detailed understanding of how the intervention was implemented in Primary Care, possible mechanisms of action and contextual factors influencing implementation. |
| Culhane-Pera (2023) | US | 50 | Education level:  High school graduate (44%)  None (22%) | 56% | 44% | 55.5 | - | 50% Latino  50% Hmong | To identify Hmong and Latino adults’ perspectives  about a mHealth-based care model for hypertension  management involving blood pressure self-monitoring,  electronic transmission of blood pressure readings, and responsive hypertension  medication adjustment by a provider team. |
| Song (2021) | Australia | 22 | Employability  Employed (50%)  Self-employed (32%)  Retired (14%)  Unemployed (5%)  Education level:  Bachelor’s Degree or higher  (41%)  College Degree (27%)  High School Graduate (23%)  Junio School Graduate (9%) | 18% | 82% | 47 | 33-73 | - | To understand the key components of an mHealth intervention to enhance self-management of hypertension in outpatients. |
| Bhandari  (2021) | Nepal | 41 | Literate:  Yes (65.8%)  No (34.2%)  Employment:  Yes (70.7%)  No (29.3%) | 51.2% | 48.8% | 48 | 30-40  (24.4%)  40-50  (26.8%)  50-60  (29.3%)  60-70  (19.5%) | 100% Nepali | To explore patients’ and providers’ acceptability of an SMS intervention for hypertension management. |
| Alzahrani  (2022) | Saudi Arabia | 21 | Education Level:  Bachelor’s Degree (57.14%) | 33.3% | 66.7% | 41-59 | 30-40  (23.8%) | - | To understand the needs of patients and providers for the design of a hypertension self-management app |
| Xiao (2019) | China | 22 | - | 50% | 50% | 60 | 43-75 | - | To understand patients’ and providers’ acceptability of a hypertension self-management mobile-phone-based platform. |
| Muehlensiepen  (2024) | Germany | 20 | Geographic context:  Urban area (65%)  Rural area (35%) | 50% | 50% | 56 | 29-80 |  | To explore patients’ experience with using an app for hypertension self-management. |
| Chu (2024) | USA | 14 | - | 50% | 50% | 57 | - | Black/African  American  (57%)  Hispanic/Latino  (14%)  White  (7%)  Other  (37%) | To explore patients’ experience with using telephone call-based remote patient monitoring embedded with team-based care for hypertension management. |
| Greer (2022) | USA | 30 | Geographic context:  Rural (100%)  Education level:  High School or less  (50%)  Higher Education  (50%)  Income annual ($)  29, 999 or less  (70%)  30,000 or above  (30%)  Insurance  Yes (93%) | 97% | 3% | 66 | - | Black/African  American  (100%) | To explore the usability and feasibility of using health apps for the management of hypertension in rural older black adults. |
| Buis (2020) | USA | 15 | Income annually ($):  <50, 000 (50%)  50,000 – 100,000 (21.4%)  >100,000 (28.6%)  Insurance:  Private (60%)  Private (40%)  Education Level:  High School or Less  (46.7%)  Some College  (26.7%)  Bachelor’s Degree  (26.7%)  Employment:  Employed (73.3%)  Retired (26.7%) | 46.7% | 53.3% | 52 | - | White  (66.7%)  Black  (20%)  Other  (13.3%) | To explore the feasibility and usability of hypertension management pharmacy-led mobile among patients and healthcare providers. |

Table S2. Critical Appraisal Skills Program (CAPS) checklist for qualitative included studies.

|  | Q1 | Q2 | Q3 | Q4 | Q5 | Q6 | Q7 | Q8 | Q9 | Q10 |
| --- | --- | --- | --- | --- | --- | --- | --- | --- | --- | --- |
| Glynn (2015) | YES | YES | YES | YES | YES | YES | YES | YES | YES | YES |
| Nichols (2019) | YES | YES | YES | YES | YES | NO | YES | YES | YES | NO |
| Baratta (2022) | YES | YES | YES | YES | YES | NO | YES | YES | YES | YES |
| Steinman (2020) | YES | YES | YES | YES | YES | NO | YES | NO | YES | NO |
| Morton (2018) | YES | YES | YES | YES | YES | NO | YES | YES | YES | YES |
| Alzahnari (2022) | YES | YES | YES | YES | YES | NO | YES | YES | YES | YES |
| Bhandari (2021) | YES | YES | YES | YES | YES | NO | YES | YES | YES | YES |
| Buis (2020) | YES | YES | YES | YES | YES | NO | YES | YES | YES | YES |
| Chu (2020) | YES | YES | YES | YES | YES | NO | YES | YES | YES | YES |
| Greer  (2020) | YES | YES | YES | YES | YES | NO | YES | YES | YES | YES |
| Muehlensiepen (2024) | YES | YES | YES | YES | YES | NO | YES | YES | YES | YES |
| Song (2021) | YES | YES | YES | YES | YES | NO | YES | YES | YES | YES |
| Xiao (2019) | YES | YES | YES | YES | YES | NO | YES | YES | YES | YES |

Table S3. Mixed Method Appraisal Tool (MMAT) for mixed-method included studies.

|  | S1 | S2 | 5.1 | 5.2 | 5.3 | 5.4 | 5.5 |
| --- | --- | --- | --- | --- | --- | --- | --- |
| Morton (2021) | YES | YES | YES | YES | YES | YES | High-quality |
| Culhane-Perta (2023) | YES | YES | YES | NO | YES | YES | Low-quality |

Table S4. Streamlined codebook of analytical-descriptive themes and exemplar quotations across all end-users.

| Streamlined Theme | Theme | Code | Definition | Example |
| --- | --- | --- | --- | --- |
| **Analytical theme: Relationship between HCPs and Patients** | | | | |
| Two-way feedback between patients-practitioners | Communication Preferences | Bidirectional Communication | Exchange of information between patients and healthcare providers, allowing both parties to send and receive health-related information. | *"It’s easier for communication about hypertension management [with my doctor] because..."* |
|  |  | Shared-decision Making | Situations where digital platforms enable collaborative decision-making processes between patients and practitioners regarding treatment plans or health management strategies. | *“Would prefer online engagement with my doctors to discuss my treatment plan when it*  *is not necessary to travel to the clinic”* |
| Tailored Communication over generalised message | Feedback on relevance | Message Relevance Feedback | Patient feedback highlighting when messages feel generic or irrelevant | *“I don’t know the messages were kinda you know, generic.”* |
|  | Contextual relevance | Timely check-in calls | Scheduled practitioner outreach triggered by specific BP readings or events | *“Called me in time and asked me what happened recently; why wasn’t my BP controlled but suddenly increased? …[it] is a wake-up call to us.”* |
|  |  | Real-world Context Alerts | Tailored warnings based on recent patient activities or events | *“Yesterday I attended a festival. After eating high salt food and meat, I am getting headache…so I think you should send information about diet as well”* |
|  | User Control | Notification control | Customizable settings allowing users to pause or stop unwanted reminders | *“I know you can’t do it in the app, but I can have, on Android I, you can, you know, force the notifications to stop.”* |
|  |  | Function optimisation on demand | Requests to eliminate redundant or unnecessary tasks in the app workflow | *“Some functions are useless. For example, I take the same drugs every day. Why do I need to upload them every day?”* |
| Sharing information with practitioners, family and friends | Social Exchange | In-App Peer Communication | Exchanging information with other users through the application’s social features | *“Share my progress with my family and my doctor and communicate with other users within the app”* |
|  | Personal network sharing | Progress Sharing with Family & Doctor | Sending personal health data and updates to close contacts and healthcare providers |  |
| **Analytical theme: Self-empowerment** | | | | |
| Generating knowledge about health status | Data visualisation and interpretation | Temporal variation insight | Recognizing patterns in health metrics across different times of day | *“When I saw the different times of days and, and how that…varied across the day…got me thinkin’ about what I was eating throughout the day…”* |
|  |  | Visualisation-induced awareness | Gaining awareness of one’s condition by viewing charts or graphs | *“I was more aware [about my health condition] as soon as I looked at the chart.”* |
|  |  | Self-interpretation | Drawing personal conclusions from observed data | *“I look how it goes and how my blood pressure level developed and draw my conclusions from that.”* |
|  | Engagement and Reflective Learning | Engagement-driven understanding | Deepening knowledge through active interaction with illness content | *“And when you engage more with your own illness, then you can, well, argue better.”* |
|  |  | Repeated exposure learning | Solidifying understanding by encountering the same information multiple times | *“When encountering the same information again [in the app], I came to understand why and how to [do it].”* |
|  |  | Reflective Prompts | Using alerts or questions to prompt self-reflection on behaviours | *“If abnormal, I was set on alert to reflect upon my own behavior. Should I get more exercise? Am I eating too much salt? Did I forget my medicine?”* |
|  |  | Threat-induced reflection | Experiencing a sense of crisis from comparative data that motivates behaviour change | *“But if you have recorded and compared yesterday’s, today’s and tomorrow’s records, a sense of crisis will push you to reflect.”* |
|  | Knowledge Formation and Correction | Education correction | Revising misconceptions based on new information | *“After reading the materials in the app, I knew I was wrong. It was time to manage hypertension.”* |
|  |  | Personal BP awareness | Gaining direct knowledge of one’s own blood pressure levels | *“I knew more about my own blood pressure.”* |
| Recognising the Impact on Managing Hypertension Risk Factors | Lifestyle Factor Awareness | Stress–BP Link Recognition | Understanding how stress at work or life events influences blood pressure | *“I was able to… distinguish a difference in my blood pressure…based on my stress at work.”* |
|  |  | Dietary Awareness | Increased attention to diet, salt intake, and food choices | *“It created an awareness within me to really pay attention to my diet, my salt intake, the types of food.”* |
|  | Trigger and Pattern Recognition | Trigger Identification | Noticing specific triggers that cause blood pressure spikes | *“I am able to understand my pressure, like what triggers it to go up and things that I eat.”* |
|  |  | Crisis-Driven Reflection | Experiencing a sense of urgency from trend comparisons that motivates behaviour change | *“But if you have recorded and compared yesterday’s, today’s and tomorrow’s records, a sense of crisis will push you to reflect. …my BP is rising. …I shouldn’t drink alcohol in a couple of days.”* |
|  | Self-Empowerment and Responsibility | Personal Responsibility Assertion | Acknowledging that managing hypertension is one’s own responsibility | *“It is my own business to manage my daily life.”* |
|  |  | Corrective Insight | Shifting beliefs and recognizing the need for active management after new information | *“After reading the materials in the app, I knew I was wrong. It was time to manage hypertension.”* |
| **Analytical Theme: Trust in Digital Technologies** | | | | |
| Reliance on healthcare professionals as credible source | Source Credibility Preference | Reliable Source Preference | Preference for information coming from trustworthy, professional sources | *"If anything comes in the mobile from a reliable source, it might have some good effect."* |
|  |  | Healthcare Professional Authority | Accepting information specifically because it comes from health professionals | *"If messages are sent by you and others like you (health professional), I will happily accept it thinking that doctor with whom I meet at the hospital."* |
|  | Trust in Professional Validation | App Trust and Information Quality | Trust in the app's information library and content quality | *"I trust the app and have already read the information, which is kept relatively concise, from the library."* |
|  |  | Professional Verification Value | Valuing apps that have content checked or verified by medical professionals | *"Your app is more trustworthy because it is targeted, specifically for hypertension...professional because [the content is] checked by doctors, unlike information [from other sources which was] just copied and pasted"* |
|  | Scepticism of Non-Professional Content | Assessment Accuracy Criticism | Questioning the accuracy of app assessments compared to professional judgment | *"Your assessment of discomfort symptoms is inaccurate and illogical."* |
|  |  | Scientific Validity Concerns | Doubting app algorithms when they contradict personal experience or professional standards | *"The assessment of BP is unscientific without considering the pressure difference...The app showed unexpectedly that my BP was normal."* |
| Insecurity about the accuracy of the clinical outcomes | Measurement Reliability Concerns | Equipment Accuracy Doubts | Questioning the precision of blood pressure monitoring devices | *"The cuff is not the most accurate. Usually before I go to the doctor, I will get a diastolic blood pressure of 100 but I have another cuff I used to use that I loaned to a friend, and I just got it back."* |
|  |  | Setting-Based Reading Variations | Recognising discrepancies between home and clinical BP measurements | *"my BP readings are always higher when I sit with the doctor but lower at home. So, the data I upload [to the app] is more accurate than that taken in the hospital."* |
|  | Clinical Decision-Making Doubts | Medication Optimisation Uncertainty | Uncertainty about app recommendations for medication adjustments | *"And with this mild or slight diastolic hypertension, it then says, 'Continue taking your blood pressure medication and consult a doctor to further optimize the medication or something in that direction."* |
|  |  | Algorithm Assessment Criticism | Questioning app algorithms that contradict personal symptoms or experience | *"The assessment of BP is unscientific without considering the pressure difference. I remember one time, I felt particularly uncomfortable, and my BP was 100/80 [mmHg]. The app showed unexpectedly that my BP was normal."* |
| Doubts about the security storage of health and personal data | Personal Information Exposure | Location and Address Sharing Concern | Worry about apps sharing home address and real-time location | *"I do not want the apps that share my home addresses and real-time location . . . . this is sensitive information . . . and I don’t want anyone to share it."* |
|  |  | Third-Party Data Sharing Fear | Fear that personal details are shared with third parties without consent | *"I am afraid if the apps share my details with other third parties, including Facebook, without taking permission from me."* |
|  | Systemic Security Risks | General Data Protection Risk | Awareness of general risks in entering health data into apps | *"Well, I do see a risk that you enter health data here, of course. This is just the data protection risk."* |
|  |  | Hacking and Unauthorised Use Anxiety | Concern that data could be hacked and misused by unknown parties | *"The data can sometimes be hacked, okay. That always raises the question of who uses this data and for what?"* |
| **Analytical Theme: Attitudes towards using digital health technologies** | | | | |
| The use of digital health technology among older population | Technology Complexity Barriers | Simplicity Requirement | Need for very basic, uncomplicated technology interfaces for older users | *"It has to be very simplistic, especially for our older patients."* |
|  |  | Basic Function Comfort | Comfort with simple phone functions but difficulty with advanced features | *"If it's just making calls, then it's pretty easy. But when I get into messages and apps and all, that kind of thing, now sometimes it gets complicated."* |
|  |  | Navigation Confusion | Uncertainty about how to proceed or navigate through digital interfaces | *"Yeah, I guess, I'm just not used to it, you know...Or like the next steps, where to go from here? Yeah, yeah, it's kinda confusing."* |
|  |  | Limited Technology Use | Using technology only for the most basic functions | *"All I use it for is calling."* |
|  | Communication Preferences | Face-to-Face Communication Preference | Preference for in-person interaction over digital communication for health matters | *"I want to talk to her face-to-face. You know, I don't want to communicate what issue is going on with my body over no phone."* |
|  |  | Age-Appropriate Messaging | Appreciation for concise, motivational messages designed for elderly users | *"I felt pretty satisfied. I think it was a concern for the elderly. The message wasn't too long...it was a motivation to us, which wasn't annoying."* |
| The use of digital health technology among young population | Technology Relationship Awareness | App Dependency Risk | Concern about becoming overly reliant on or attached to digital health applications | *"Maybe that you get too attached to the app. But that's always the case with things like this. But I tend to forget that."* |
| Motivation to continue self-management | Support and Encouragement | Reminder Support | Receiving reminders for medication, exercise, or healthy behaviours | *"I need the app that keep reminding me to do my exercise and food suitable for BP."* |
|  |  | Gratitude for Encouragement | Feeling grateful for positive reminders and motivational messages | *"Every time I received the reminder, I felt grateful."* |
|  |  | Achievement Recognition | Feeling encouraged by recognition of completed tasks or achievements | *"If I walk up to 5,000 steps, it will pop up a message saying, 'Congratulations! You have reached 5,000 steps.'"* |
|  | Self-Monitoring and Feedback | Data-Driven Motivation | Motivation from seeing progress in health data or records | *"By seeing the curve of my BP recording being flattened. This gives me the motivation to keep going."* |
|  |  | Feedback Desire | Wanting feedback and acknowledgement of efforts | *"It would be nice that my effort could be faithfully recorded by your app. ...But now, look at these records. I have the strength."* |
|  |  | Clarity of Criteria | Needing clear criteria for performance and progress to stay motivated | *"I don’t know the criteria for scoring and ranking the performance, so I’m not motivated because I don’t know what to do next."* |
|  | Social and Educational Factors | Peer Comparison | Motivation from comparing progress with others | *"Seeing that someone was ahead of me, I made a firm decision that I had to catch up with him or her."* |
|  |  | Educational Empowerment | Gaining motivation from understanding self-management through educational content | *"Now, I know how to self-manage [hypertension] well.”* |
|  | Goal Setting and Consistency | Goal Setting | Setting personal health goals to manage blood pressure and nutrition | *"To control my nutrition in order to control my blood pressure, set goals, and reminder for healthy food"* |
|  |  | Consistency Challenge | Struggling to maintain consistent self-management due to personal barriers | *"I take my blood pressure twice a week, but I am supposed to take it twice a day. ...Nothing about the program can help me be more consistent. It’s just me."* |
| **Analytical Theme: Usability of digital health technologies** | | | | |
| Technological difficulties with digital tools | Basic Accessibility | Accessibility via Simple Tech | Ease of accessing information with basic mobile technology | *"Can very easily see the information in mobile without any difficulties and there is no need to have expensive smartphone for simple SMS."* |
|  | Device and System Reliability | Device Pairing Issues | Problems syncing devices (e.g., phone and blood pressure monitor) | *"Phone would unsync from the blood pressure. (Um-hm) And it would take several tries. Even though it said it paired, it did not pair."* |
|  |  | Connectivity and Offline Issues | Occasional loss of connection or going offline | *"Occasionally, it may fall offline."* |
|  | Account and User Management | Login and Account Access Problems | Difficulties logging into accounts, leading to loss of user confidence | *"Sometimes I can’t log into my account. You know, users will lose confidence in your product in this case."* |
| Social Support | Family and Peer Support | Family Involvement | Sending information to family members to support patients | *"If we could send the message to the family member of the illiterate patients, they will get the information."* |
|  |  | Intergenerational Assistance | Help from younger family members, such as grandchildren | *"Yes, my grandchild helps me out with mine, with everything from my medication to his homework, to everything."* |
|  |  | Peer Communication | Ability to communicate with other users within the app | *"And communicate with other users within the app."* |
|  | Emotional and Motivational Support | App as a Companion | The app providing a sense of companionship and daily support | *"It [the app] is like a kind of companion, like a sparring partner basically."* |
|  |  | Recognition and Encouragement | Receiving motivational messages or recognition for achievements | *"if I walk up to 5,000 steps, it will pop up a message saying, 'Congratulations! You have reached 5,000 steps...'"* |
| Improving the social aspect of care | Remote and Convenient Care | Online Doctor Engagement | Preference for online discussions with doctors to avoid unnecessary clinic visits | *"Would prefer online engagement with my doctors to discuss my treatment plan when it is not necessary to travel to the clinic."* |
|  |  | Accessibility and Convenience | Ease of accessing care without travelling to hospitals or waiting in crowded spaces | *"[I] do not have to run to the hospital. It has always been annoying to go to the hospital to seek medical treatment. We have to wait in a crowded space for a long time, and the queue is often quite long."* |
|  | Familiar and Comfortable Settings | Comfortable Monitoring Environment | Appreciation for monitoring health in familiar, non-clinical settings | *"I liked the most about the program was that it was in an environment that wasn’t at a doctor’s office… I could check my blood pressure in a usual state, either work, or home, or whatever."* |
| **Analytical Theme: Impact post-intervention** | | | | |
| Medication Adherence | Digital Reminder Systems | Medication Reminder Support | Using technology to help remember to take medications | *"Helped me remember to take my medication for one thing."* |
|  |  | Alarm-Based Adherence | Using alarms or alerts to prompt medication taking | *"Just won't forget it. I used to forget about (taking medication on time). Now once the alarm rings, I will take the medications."* |
|  |  | Timely Medication Reminders | Receiving consistent reminders to take medicine at the correct time | *"[The app] always reminds me to take medicine on time."* |
| Sustained Engagement | Provider Integration | Healthcare Provider Data Access | Enabling doctors to access patient information through digital platforms | *"Number to allow doctors to access patients' information and link to their electronic file."* |

Table S5. Facilitators and Barriers with Exemplar Quotations

| **Facilitators and barriers to end-users' utilisation of digital self-management intervention for hypertension** | | | | | |
| --- | --- | --- | --- | --- | --- |
| Analytical Theme | Descriptive Theme | Facilitator | Example quotations | Barrier | Example quotations |
| Patients’ experience | | | | | |
| Relationship with practitioners | Two-way feedback between practitioners and patients | Improved communication and relationship with practitioners | *“So I would like to be able to …screen save it ….scan it…and save it and bring it up and show it to (my doctor)”*^1^ | Fear of losing face-to-face consultations with practitioners | *“I want to talk to her face-to-face. You know, I don’t want to communicate what issue is going on with my body over no phone.”* ^2^ |
|  | Tailored communication over generalised messages | Improved consultation process and discussion | *“If specific information (such as this much salt is allowed) will be sent for us, definitely it would help for changing our habits”*^3^ |  |  |
|  | Sharing information with practitioners, family and friends | Ability to share BP readings with a support network | “*It’s like having a doctor at home and they’re seeing things in real time. It would be relieving for patients.”* ^4^ | Fear of opening confrontation with practitioners | *“I just have a feeling that she would feel that she was being undermined, you know, I just, you know I would”*^1^ |
| Self-empowerment | Generating knowledge about health status | Enhanced self-management skills | *“I look how it goes and how my blood pressure level developed and draw my conclusions from that.”* ^5^ | Unrealistic expectations of lifestyle changes | *“It didn't really relate to me as I don't smoke”*^6^ |
|  |  |  |  | Lack of tailoring messages and educational resources | *“I’m thinking about why my blood pressure has gone up. I can’t think why.”*^7^ |
|  | Recognising the impact on managing hypertension risk factor | Improved understanding of health status | *“When encountering the same information again [in the app], I came to understand why and how to [do it]”*^8^ | Insecurity about the ability to maintain engagement and use of digital tool | *“The blood pressure cuff is probably the easiest [remote monitoring digital health tool] to use but there are several steps involved”*^9^*.* |
| Trust in digital technologies | Reliance on healthcare professionals as a credible source | Trust in healthcare providers’ guidance | *“If anything comes in the mobile from a reliable source, it might have some good effect”*^3^*.* | Lack of face-to-face consultation | *“I wouldn’t trust (my blood pressure) for 6 months without going to the doctor”* ^1^*.* |
|  |  |  |  | Lack of self-trust in engaging with behaviour-change content | *"Because of my good lifestyle has gone fallen by the wayside”*^1^. |
|  | Insecurity about the accuracy of the clinical outcomes |  |  | Lack of reassurance on BP reading and educational sources | *“The assessment of BP is unscientific without considering the pressure difference”*^8^*.* |
|  | Doubts about the security storage of health and personal data |  |  | Lack of reassurance on personal data management | *“I am afraid if the apps share my details with other third parties”*^10^. |
| Attitudes toward using digital technologies | The use of digital health technology among older populations |  |  | Lack of access to a phone | *“I don’t know how to use the phone!”*^11^ |
|  |  |  |  | Reduced digital literacy | *“It has to be very simplistic”*^12^ |
|  |  |  |  | Lack of user-centred design | *“I get into messages and apps and all, that kind of thing, now sometimes it gets complicated”*^2^. |
|  | The use of digital technology among younger population | Enhanced confidence in managing BP | *“It looks easy for the younger educated people”*^4^. | Lack of tailoring messages and educational information | “*…it has to be individually tailored.”*^1^*.* |
|  |  | Increased acceptability in remote control of BP | *“I downloaded it and, yes, it was also easy to use”*^5^*.* | Increase of anxiety | *“Maybe that you get too attached to the app”*^5^. |
|  | Motivation to continue self-management | Received motivational messages when meeting the goal | *“For me, what helped was the messages that say you have done well”*^6^. |  |  |
|  |  | Enabled the generation of knowledge | *“I went through the educational material little by little”*^8^ |  |  |
|  |  | Received educational messages and reminders of medication | *“I need the app that keep reminding me”*^10^*.* |  |  |
|  |  | Enabled an action-planning strategy | *“If you do yourself a small plan you are achieving it and then that gives you motivation and stuff”*^1^. |  |  |
| Usability of digital technologies | Technological difficulties with digital tools | Enabled training of digital technical support | *"Participants suggested future training include recommendations on how to trouble technology challenges"*^6^. | Depending on digital technology literacy | *“I don’t think that I am smart enough to use the cuff and the smartphone”*^1^ |
|  |  |  |  | Troubleshooting issues | *“Users will lose confidence in your product in this case”*^8^. |
|  | Social support | Tailoring cultural settings | *“The app is my sparring partner because it also communicates with me”.*^5^ | Depending on friends and family | *“I have children and they haven't attempt [sic] to help me”*^2^*.* |
|  | Improving the social aspect of care | Increasing access to healthcare in rural areas | *“[I] do not have to run to the hospital”.*^8^ |  |  |
|  |  | Reducing patients’ travelling time | *“It can be taken and used everywhere”*^8^*.* |  |  |
| Impact post-intervention | Medication adherence | Sustained behaviour changes practices | *“Now once the alarm rings, I will take the medications”*^8^*.* | Difficulty maintaining long-term engagement with digital tools | *“We would be given these machines and smartphones for like 6 months or more instead of the 3 months”*^6^*.* |
|  | Sustained engagement | Maintaining the EHR and BP measures synchronised | *“Proper record-keeping is essential”*^3^ |  |  |
| **Caregivers’ experience** | | | | | |
| Impact of digital technologies on hypertension care | Improving the social aspect of hypertension care | Increasing confidence in providing care. | *“Turned my wife into a nurse”*^6^*.* |  |  |
|  |  | Reducing time on travelling to health facilities | *"Even if our BP is high we wouldn't know unless we come to the clinic but that wasn't the case when we had the machines"*^6^*.* |  |  |
|  | Improving the clinical aspect of hypertension care | Enabling monitoring of medication and clinical measures | *“But without the machine, it is difficult to stick to a time”*^6^*.* |  |  |
| Usability of digital technologies | Improving digital literacy | Enabling training in digital technical support | *“Some patients may be literate but technological literacy is also another thing“*^6^*.* | Time-consuming | *“Was unable to be present consistently during the windows of time each day”*^6^*.* |
| **Healthcare providers’ (HCPs) experience** | | | | | |
| Relationship with patients | Sharing information with patients | Improved consultation process and discussion | *“We can send one SMS to millions of people in a short period”*^3^*.* | Enabling a standardised communication | *“I’ve just used your templates and that was fine”*^13^. |
|  |  | Improved communication and relationships with patients | *“The management model of daily monitoring increased the communication time between the patients“*^3^. |  |  |
| Impact of digital technologies on hypertension care | Improving clinical care | Enabling remote control of BP readings | *“It saves a lot of time when you’re meeting with patients cause you have all that information ahead of time”*^9^. | Enabling multiple daily reads of BP in electronic health records | *“You’ve got a plan and now that’s changing and now do I have to make another three-point plan?”*^13^ |
|  |  | Enabling visual tools for BP readings | *“It saves a lot of time when you’re meeting with patients cause you have all that information ahead of time”*^9^. | Lack of flexibility in implementing the BP reading plan | *“There is not even a good record-keeping system here in our hospital, and no one is going to record as we don’t have time”*^3^ |
|  |  |  |  | Lack of centred-user design | *“I wish that they were more accurate; more consistent”*^12^*.* |
|  | Improving clinical aspect care among patients with limited access to healthcare | Facilitating escalation of medication | *“You don't need to contact the patient, you just do the prescription”*^13^*.* | Medication inertia in escalating prescription | *“I'm not happy to escalate it”*^13^*.* |
|  |  | Enabling tailored voice messages | “*And make it relative”*^1^*.* |  |  |

**Supplementary Text S1: Search strategy**

PubMed Database (Search date: February 2025)

((Young adults*[tiab] OR Young*[tiab] OR Age*[tiab] OR ethnicities*[tiab] OR ethnicity*[tiab] OR ethnic*[tiab] OR minority*[tiab] OR minorities*[tiab] OR patients*[tiab] OR black*[tiab] OR blacks*[tiab] OR asian*[tiab] OR asians*[tiab] OR african*[tiab] OR BME*[tiab] OR ethnically minorities*[tiab]) AND ("Hypertension"[Mesh] OR hypertensi[tiab] OR prehypertensi*[tiab] OR blood pressure[tiab] AND ("Providers"[mesh] OR "Nurses"[mesh] OR physician*[tiab] OR provider*[tiab] OR clinician*[tiab] OR general physician*[tiab] OR primary care*[tiab] OR general physicians[tiab] OR primary physicians[tiab]) AND  (“Mobile Applications”[Mesh] OR “Cell Phones”[Mesh] OR “Computers, Handheld”[mesh] OR ((app[tiab] OR apps[tiab] OR application*[tiab] OR technology[tiab] OR platform*[tiab] OR computer program*[tiab] OR software[tiab]) AND (smartphone*[tiab] OR phone[tiab] OR phones[tiab] OR tablet*[tiab] OR handheld*[tiab] OR iphone*[tiab] OR ipad*[tiab] OR android*[tiab])) OR mobile app*[tiab] OR mobile technolog*[tiab] OR “mobile device*[tiab] OR mobile compu* OR wearable[tiab])" OR (“Desicion Support Systems, Clinical”[Mesh] OR decision support*[tiab] OR remote monitor*[tiab] OR televisi*[tiab] OR telehealth*[tiab] OR mobile health*[tiab] OR ehealth*[tiab] OR mhealth*[tiab] OR digital health*[tiab] OR connected health*[tiab]) OR (“Health Records, Personal”[Mesh] OR “Electronic Health Records”[Mesh] OR “Medical Records Systems, Computerized”[Mesh] OR personal health record*[tiab] OR personal medical record*[tiab] OR personally controlled health record*[tiab] OR Personal Electronic Health Record*[tiab] OR Computerized Patient Record*[tiab] OR computarized record*[tiab] OR electronic health record*[tiab] OR electronic medical record*[tiab] OR emr[tiab] OR emrs[tiab] OR phr[tiab] OR phrs[tiab] OR pchr[tiab] OR pchrs[tiab] OR ehr[tiab] OR ehrs[tiab] OR automated health record*[tiab] OR automated medical record*[tiab]))

**Supplementary Text S2: Search strategy**

PsycINFO Database (Search date: February 2025)

TX(Young adults OR Young OR Age OR ethnicities OR ethnicity OR ethnic OR minority OR minorities OR patients OR black OR blacks OR asian OR asians OR african OR BME OR ethnically minorities) AND TX(" Hypertension" OR prehypertension OR blood pressure) AND  TX(“Mobile Applications”OR “Cell Phones” OR “Computers, Handheld” OR ((app OR apps OR application OR technology OR platform OR computer program OR software) AND (smartphone OR phone OR phones OR tablet OR handheld OR iphone OR ipad OR android)) OR mobile app OR mobile technology OR “mobile device* OR mobile computer OR wearable) OR (“Decision Support Systems, Clinical” OR decision support*[tiab] OR remote monitor*[tiab] OR televisi*[tiab] OR telehealth*[tiab] OR mobile health OR ehealth OR mhealth OR digital health OR connected health) OR (“Health Records, Personal” OR “Electronic Health Records” OR “Medical Records Systems, Computerized” OR personal health record* OR personal medical record OR personally controlled health record OR Personal Electronic Health Record OR Computerized Patient Record OR computerized record OR electronic health record OR electronic medical record OR emr OR emrs OR phr OR phrs OR pchr OR pchrs OR ehr OR ehrs OR automated health record OR automated medical record))

**Supplementary Text S3: Search strategy**

Cochrane Central Databases (Search date: February 2025)

#1 ("hypertension" OR "high blood pressure" OR "prehypertension”):ti,ab

#2 (("cell phone" OR "application" OR "technologic" OR "platform" OR "computer program" OR "software" OR "phone" OR "tablet" OR "handheld" OR "wearable" OR "televis*" OR "remote control" OR "telehealth" OR "telehealthcare" OR "ehealth" OR "mhealth" OR "digital health" OR “connected health”): ti,ab (OR (“Health Records” OR “Electronic Health Records” OR “Medical Records Systems” OR “personal health record” OR “personal medical record” OR “personally controlled health record” OR “Personal Electronic Health Record” OR “Computerized Patient Record” OR “computerised record” OR “electronic health record” OR “electronic medical record” OR “emr” OR “emrs” OR “phr” OR “phrs” OR “pchr” OR “pchrs” OR “ehr” OR “ehrs” OR “automated health record” OR “automated medical record”)): ti,ab

#3("prevention" OR "management" OR "primary-care" OR "Providers" OR "Nurses" OR **"**physician**"** OR **"**provider**"** OR **"**clinician**"** OR **"**general physician**"** OR “primary care” OR “general physicians” OR “primary physicians”): ti,ab

#1 and #2 and #3

**Supplementary Text S4: Search strategy**

Web of Science Core Collection Database (February 2025)

#1 TS= ((adult OR patient))

#2 TS= ((Hypertension OR prehypertension OR high blood pressure))

#3 TS= ((mobile application OR App* OR Cell phones OR Technolog* OR Platform OR  Phone OR Computer program OR smartphone OR software OR Tablet OR iPhone OR iPad OR Android OR  Mobile technology OR wearable OR  televi* OR Telehealth OR Digital Health OR eearth OR mealth OR Connected health))

#4 TS= ((Providers OR Nurses OR Physician OR Provider clinician OR General physician OR Primary care OR  Primary physician))

#5 TS= ((Infection OR Communicable disease))

 #1 and #2 and #3 and #4 not #5
