# Supplementary material for: Barriers and facilitators to digital health tool adoption for hypertension management: systematic review of qualitative studies
Source: BMJ Open. 2026 Jun 12;16(6):e116004. doi: 10.1136/bmjopen-2025-116004 (PMC13289156; doi:10.1136/bmjopen-2025-116004)
Supplement: Supplementary file 2 [file bmjopen-2025-116004.reviewer_comments.pdf]

PEER REVIEW HISTORY

BMJ Open publishes all reviews undertaken for accepted manuscripts. Reviewers are asked to complete a checklist review form and are provided with free text boxes to elaborate on their assessment. These free text comments are reproduced below.

ARTICLE DETAILS

Title (Provisional)

Barriers and facilitators to digital health tool adoption for hypertension management: systematic review of qualitative studies

Authors

Motta, Emily; Riley, V A; Ellis, N J; Mankoo, Aman; Gidlow, Christopher J

VERSION 1 - REVIEW

**Reviewer**	**1**
**Name**	**Oseni, Tijani**
**Affiliation**	**Edo State University Uzairue, Family Medicine**
**Date**	**19-Feb-2026**
**COI**	**None**

The systematic review looked at the barriers and facilitators to digital health tool adoption for hypertension management.

The abstract is clearly stated and is a summary of the work.

The introduction was properly written.

The methodology was rigorous and the reporting followed PRISMA and the review was registered with PROSPERO.

The results were well reported and discussed adequately.

The references were relevant and up to date.

However, there are some minor issues to be addressed:

1. Page 3, Line 83: Under search strategy you said the study was conducted between January 2015 to January 2025. Did you mean you searched studies conducted between January 2015 to January 2025.

2. Page 3, Line 89: In your selection criteria why did you exclude hypertensive patients

3. Some grammatical errors that need correction, for example, on page 9, line 271:, the statement .the review follows ... should have read .. the review followed... Go through the manuscript and correct all grammatical errors.

**Reviewer**	**2**
**Name**	**Alhenc-Gelas , Francois**
**Affiliation**	**INSERM, U1138 CRC**
**Date**	**25-Feb-2026**
**COI**	**None**

The topic of this study, acceptability of digital health tools in hypertension care, is of both clinical and public health relevance. The study is well presented. It has been conducted with proper methodology, within the limit of the defined premises: qualitative assessment of outcomes, no formal documentation of reproducibility. Outcomes are based in a part on author subjectivity but the thematic identified and the recommandations associated are generally sound. The study may be a useful addition to a topic of digital health tools in hypertension care, that has seldom been reviewed with this focus.

A few issues may be addressed for further improving the study:

-RCT (n-44, figure 1) were excluded. This should be commented.

-Distinction between caregiver (practician nurses?) and health providers may not be familiar to readers in all parts of the world. This can be further explained.

-The issue of cost is not addressed. This is not relevant to sponsored trials but may become a problem for use in the general hypertensive population and cannot be completely ignored in a work focused on feasibility.

-The main issue with digital health tools beyond acceptability is efficacy and effect on blood pressure control in population of patients wit hypertension. This is not the focus of the review but is addressed, albeit relatively loosely, in the last paragraph of the result section. Reference to properly conducted efficacy studies can be made here.

**Reviewer**	**3**
**Name**	**Dawes, Martin**
**Affiliation**	**University of British Columbia, Family Practice**
**Date**	**27-Feb-2026**
**COI**	**I am part of a CIHR funded RCT on patient BP monitoring with upload of data too the hospital clinical team.**

Review bmjopen-2025-116004

This review aims to synthesise qualitative evidence to identify barriers and facilitators, and the key factors that impact the adoption, acceptance, and engagement with digital health interventions for hypertension self-management.

They identified several themes: usability, design, and relevance of social support – meaning having others available to help the patient use the technology.

I am anxious that readers may see this and say that they knew that. So what is new? It is really well-done study so deserves to be published but it misses an “aha” element. I have suggested one idea at the end and there may be other angles that could be used to increase the impact of the findings.

Abstract is clear and well written.

Introduction is clear but examples and rates of adoption would help non expert audiences. A picture of one would be great.

Search strategy. There may be a lot of grey literature about these tools that might yield further information.

Selection criteria good.

CASP appraisal appropriate

Thematic analysis described

Results are described appropriately

The themes appear to be substantiated by the text, and they resonate with experience.

The discussion is interesting but I am interested in this. For a general audience I am not so sure. It would be good to see how many of these themes might be expected based on recent studies of other technologies. What are the surprising themes? Which are the most important (based on the qualitative data)? There are trade-offs of device clinical utility versus usability. Did this appear? In this situation many might perceive BP as low importance compared to other conditions. Is that a factor?

Overall, all the themes have been discussed for other technologies. Perhaps it may be more interesting to review the devices and look at how many intentionally addressed these themes in their design. Exploration of the regulatory application of one or two recent devices, or even a look at regulatory application templates, might show that regulatory bodies are in sync/or not in sync with these themes.

VERSION 1 - AUTHOR RESPONSE

Reviewer 01

1. Page 3, Line 83: Under search strategy you said the study was conducted between January 2015 to January 2025. Did you mean you searched studies conducted between January 2015 to January 2025.

   Response: I appreciate you pointing out this lack of clarity. We have revised the sentence to clarify that the date range refers to the publication period of the included studies (January 2015 to January 2025), rather than the duration of the search process itself. The search was executed in February 2025. Manuscript Change (Page 3, Line 82): "A systematic computerised search [...] was conducted in February 2025 to identify studies published between January 2015 and January 2025."

2. Page 3, Line 89: In your selection criteria why did you exclude hypertensive patients.

   Response: I appreciate this observation. Patients with hypertension (high BP) were the primary population included in this review. We have now reformatted the "Selection Criteria" section into a structured list to more clearly distinguish between our inclusion of hypertensive adults and the specific exclusion of pregnancy-related hypertension, for which the original wording may have been ambiguous. Manuscript Change (Page 3, Line 89): The section has been revised to state: "Inclusion Criteria: participants with a primary diagnosis of essential hypertension...".

3. Some grammatical errors that need correction, for example, on page 9, line 271:, the statement .the review follows ... should have read .. the review followed... Go through the manuscript and correct all grammatical errors.

   Response: Thank you for the observation. In the revision version grammatical errors have been corrected. Manuscript changes (page 8, line 280): "the review followed"; (page 9, page 284): "Our findings aligned…"; (page 8, line 286): "These findings also emphasised..."; (page 9, 289): "…significantly influenced the effective…"; (page 9, line 293): "Our findings suggested…"; (page 9, line 304): "Usability was another critical factor…"; (page 9, line 307): "…community members played…"; (page 9, line 330): "…digital tools remained…"; (page 9, line 333): "Our review suggested…"; (page 10, line 340): "Our findings suggested…"; (page 10, line 343): "For caregivers, DHIs held…"

Reviewer 02

1. RCT (n-44, figure 1) were excluded. This should be commented.

Response: We have added a comment to the Results section to clarify this. (Page 4, Line 114-117) “As illustrated in the PRISMA flow diagram, 44 RCTs were excluded during the full-text screening phase. These studies were excluded because they focused on the quantitative clinical effectiveness of DHIs rather than the qualitative experiences, barriers, or facilitators of adoption”

2. Distinction between caregiver (practician nurses?) and health providers may not be familiar to readers in all parts of the world. This can be further explained.

   Response: We appreciate the need to consider regional differences in terminology. In our study, "caregivers" refers exclusively to informal caregivers, such as family members and friends, rather than professional nursing staff. To avoid potential confusion for an international audience, we have revised the manuscript to use the term "informal caregivers" throughout and have added an explicit definition in the Methods section. We added a definition to the "Selection Criteria" section: "We define caregivers as informal, unpaid individuals (e.g., family or friends) providing support, distinct from professional healthcare providers.

3. The issue of cost is not addressed. This is not relevant to sponsored trials but may become a problem for use in the general hypertensive population and cannot be completely ignored in a work focused on feasibility.

   Response: We agree that the costs for smart devices are a barrier to access and adoption, and have now added a paragraph in the discussion section to reflect on the potential impact on scalability of digital tools. Manuscript changes: “Furthermore, the economic feasibility of digital health adoption emerged as a pivotal factor for real-world implementation. While clinical trials often mitigate financial barriers by providing free equipment and connectivity, our analysis suggested that for the general hypertensive population, the costs of smart devices and recurring mobile data charges remain potential barriers to adoption.”

4. The main issue with digital health tools beyond acceptability is efficacy and effect on blood pressure control in population of patients with hypertension. This is not the focus of the review but is addressed, albeit relatively loosely, in the last paragraph of the result section. Reference to properly conducted efficacy studies can be made here.

   Response: We thank the reviewer for this suggestion. We agree that providing a quantitative context for the efficacy of these tools strengthens the clinical relevance of our qualitative findings. We have revised the final paragraph of the Results section (under "Impact of digital technologies on hypertension care") to include data from a recent systematic review and meta-analysis. Manuscript change: “While this review focuses on qualitative experiences, these perceptions are supported by quantitative evidence demonstrating the clinical efficacy of DHIs.

Recent meta-analyses of RCTs indicate that digital interventions significantly reduce office systolic blood pressure by 3.21-4.05 mmHg.”

Reviewer 03

The discussion is interesting but I am interested in this. For a general audience I am not so sure. It would be good to see how many of these themes might be expected based on recent studies of other technologies. What are the surprising themes? Which are the most important (based on the qualitative data)? There are trade-offs of device clinical utility versus usability. Did this appear? In this situation many might perceive BP as low importance compared to other conditions. Is that a factor?

Perhaps it may be more interesting to review the devices and look at how many intentionally addressed these themes in their design.

Exploration of the regulatory application of one or two recent devices, or even a look at regulatory application templates, might show that regulatory bodies are in sync/or not in sync with these themes.

Response: We thank the reviewer for these insightful reflections. We have addressed these comments by integrating a new "Future Implications" paragraph directly into the Discussion (Page 10, Line 314-329). By doing so, we have expanded our interpretation to reflect on several of your key points without deviating from the qualitative scope of this review. Specifically, we have addressed the utility vs usability trade-off by discussing how the burden of high-quality monitoring can lead to disengagement; reflected on the asymptomatic nature of hypertension by acknowledging that hypertension is often perceived as a lower priority due to its lack of immediate symptoms; highlighted the role of caregivers by emphasising that the informal caregivers is a primary, though often overlooked, facilitator of long-term care; discussed regulatory alignment by commenting on the need for regulatory templates to move beyond technical usability and incorporated the sociocultural realities of the patient-caregiver relationship.

We believe this addition provides the impact factor requested for a general audience while maintaining the thematic flow of the discussion.

VERSION 2 - REVIEW

**Reviewer**	**3**
**Name**	**Dawes, Martin**
**Affiliation**	**University of British Columbia, Family Practice**
**Date**	**20-Apr-2026**

COI

Thank you for addressing the reviewers points. Basically this is a damning review and a call for better design that resonates with my cliclnicalical experience. So this may be a review affected by my own bias. Despite that bias I see the work as rigorous and impartial as it could be and the results are likely to be an accurate description. The work is important and will raise questions about why a technology that has ben in place for so many years still is sub-optimal.
